# Exploring the usability of a videophone mock-up for persons with dementia and their significant others

**DOI:** 10.1186/1471-2318-14-49

**Published:** 2014-04-16

**Authors:** Inga-Lill Boman, Stefan Lundberg, Sofia Starkhammar, Louise Nygård

**Affiliations:** 1 Department of Neurobiology, Care Sciences and Society, Division of Occupational Therapy, Karolinska Institutet, 141 83 Huddinge, Sweden; 2 Department of Rehabilitation Medicine, Danderyd University Hospital, 182 88 Stockholm, Sweden; 3 Royal Institute of Technology, School of Technology and Health, 136 40 Handen, Sweden; 4 Karolinska University Hospital, 171 76 Solna, Sweden

**Keywords:** Assistive technology, Communication, Dementia, Touch screen

## Abstract

**Background:**

Persons with dementia might have considerable difficulties in using an ordinary telephone. Being able to use the telephone can be very important in order to maintain their social network, getting stimulation and for reaching help when needed. Therefore, persons with dementia might need an easy-to-use videophone to prevent social isolation and to feel safe and independent. This study reports the evaluation of the usability of a touch-screen videophone mock-up for persons with dementia and their significant others.

**Methods:**

Four persons with dementia and their significant others tested the videophone mock-up at a living laboratory. In order to gain knowledge of the participants’ with dementia ability to use their own computers and telephones, interviews and observations were conducted.

**Results:**

Overall, the participants had a very positive attitude towards the videophone. The participants with dementia perceived that it was useful, enjoyable and easy to use, although they initially had difficulties in understanding how to handle some functions, thus indicating that the design needs to be further developed to be more intuitive.

**Conclusions:**

The findings suggest that the videophone has the potential to enable telephone calls without assistance and add quality in communication.

## Background

It is well-known that persons with dementia are likely to have considerable difficulties in using communication technology such as an ordinary telephone or cell phone [1-3]. Being able to use the telephone can be very important for many purposes such as maintaining social networks, getting stimulation, and for reaching help when needed [2,4]. The importance of developing user-friendly and less complex communication technology for persons with dementia has been increasingly recognised [4-6]. In the last decade various communication technology solutions such as easy to use telephones or computer-aided telephone systems [5,7-9] have been developed to support persons with dementia in using a telephone on their own [10]. However, when designing for persons with dementia, it is important to consider the users’ increasingly limited ability to learn how to use new things [1]. Research has suggested that the process of learning how to use a piece of technology in persons with dementia is inseparable from the very use of it [6]. To facilitate introduction of new technologies, it is important that the design is user-friendly, intuitive, familiar, and as similar as possible to what the user is familiar [6,11].

Persons with dementia might not only have difficulty handling an ordinary telephone; they might also have difficulty visualising the person they wish to talk to when she or he is not present as well as to hear, interpret, and understand what is said in the conversation [12,13]. Moreover, talking on the telephone might be experienced as an abstract action that makes it difficult to relate to the person spoken to [2,3]. Therefore, a videophone might be an appropriate communication tool for persons with dementia to help them demonstrate their ideas and to understand what is said in a conversation with gestures, signs and body language, and to feel safe and secure [9] while they interpret the other person’s message [14]. Earlier research has emphasised the importance of developing communication technology for persons with dementia for maintaining the social network and for reaching help when needed [4,10,14-16]. To address this need, the researchers set out to develop an easy-to-use videophone based on the principles of Inclusive Design that includes the target user in the design process [17]. The method comprises four phases. The first three phases have been reported in two earlier articles [1,12]. First, the need of a videophone and the users’ potential for managing a videophone was examined. This led to detailed identification of cognitive, physical, and psychosocial challenges that persons with dementia are likely to have when using an ordinary telephone or videophone. The researchers also investigated all similar products available at that time, and they found that none of them was sufficiently easy to use and none met the requirements of persons with dementia. Based on this finding, a requirement specification was formulated [1]. In the next phase, a design concept was developed that was evaluated in focus groups of persons with dementia, their significant others, and professionals. The focus groups were also conducted to gain a better understanding of the users’ actual need for a videophone. The findings showed that it is important that the videophone is flexible and easy to use and understand, and it should not be identified as an assistive technology. Moreover, the videophone was acknowledged to have potentials to enable calls without assistance and add quality in communication. There was one evident difference between the viewpoints of the three different samples in the focus groups. The significant others wanted to be able to monitor the persons with dementia when they were away from home in order to check that everything was well; however, the persons with dementia explicitly underscored that they did not want to be monitored. The professionals acknowledged the ethical aspects of tracking and monitoring persons with dementia, and they pointed out that it is important that technology is not misused [12].

Subsequently in the fourth phase, a mock-up of the videophone was developed. This article presents the first evaluation of the usability of that mock-up. Usability is defined as how efficient the mock-up was to support the participants with dementia to carry out the required tasks in the test [18].

The aim of this study was to evaluate the usability of a videophone mock-up for persons with dementia and their significant others. Two questions were formulated:

1. How did the participants perceive that the features of the videophone mock-up could support them to carry out the required tasks in the test session?

2. How did the participants perceive that the features of the videophone mock-up could meet their requirements?

## Methods

### Participants

Persons with dementia and their significant others were recruited through an investigation memory unit. Potential participants were invited by telephone and by a letter with information. Inclusion criteria were: being diagnosed with dementia; being able to participate in the combination of interviews and observations; being willing to test the videophone mock-up in a living laboratory at the Royal Institute of Technology; and being a significant other willing to participate. The persons with dementia chose significant others; they could be a relative or a close friend that had good insight into the everyday life of these persons. After persons who agreed to participate gave their verbal consent and times were scheduled for an initial interview, an observation session was scheduled at home and a test of the videophone mock-up at the living laboratory was scheduled. If the participants wanted help to book a taxi to the test session, the researchers provided assistance. Four persons with dementia and their significant others were included in the study (see Table 1 for demographics of the participants with dementia and their significant others).

**Table 1:** Demographics of the participants with dementia and their significant others

**Participants**	**Age**	**Occupation**	**Time since dementia diagnosis**
1. Ingrid	66	Retired (preschool teacher)	6 years
Ingrid’s husband	67	Semi-retired (engineer)	
2. Sven	74	Retired (clerk)	3 years
Sven’s daughter	43	Rehabilitation counsellor	
3. Maria	71	Retired (teacher)	4 years
Maria’s friend	69	Retired (librarian)	
4. Arne	69	Retired (constructor)	6 years
Arne’s wife	72	Retired (administrator)	

### The design concept

Initially, a first preliminary design concept was developed in collaboration with two master students. The iterative process was then continued, and new versions of the design concept were developed based on the requirement specification that was formulated in our first study of the videophone [1] and with continuous discussions in the research group. The final design concept had a touch screen with pictures present on the screen, a camera on the top of the screen, a support holder to keep it standing, and a handset. In order to make a call, the user should pick up the handset and then the screen will automatically show the contact list. To make a call, the user should press the picture of the contact that he or she would like to call. For incoming calls, visual feedback is given on the screen with a picture and name of the caller. In order to answer, the user has to pick up the handset to receive the call. During the video chat, the screen will only show the video of the person that the user is talking to. To finish the call, the user has to hang up the handset. The video call will automatically shut down if the user forgets to hang up.

### The mock-up

When the mock-up was to be set up, the findings from the focus groups [12] provided insight on how this should be done. For example, the participants recommended that the SOS button on the top of the screen should be an icon in the contact list in order to be easier to find and use. Furthermore, they recommended red frames around the SOS-icon and the icon for one selected person whom the user would likely call if they needed help in a situation that was not an emergency. The participants in the focus groups had different opinions on whether there should be a handset. Therefore, the decision was made to have a handset, but it was also possible to use the mock-up without using the handset. The mock-up needed to have three functions: receiving a call, making a call to a person on the contact list, and an emergency call.

**Figure 1:**
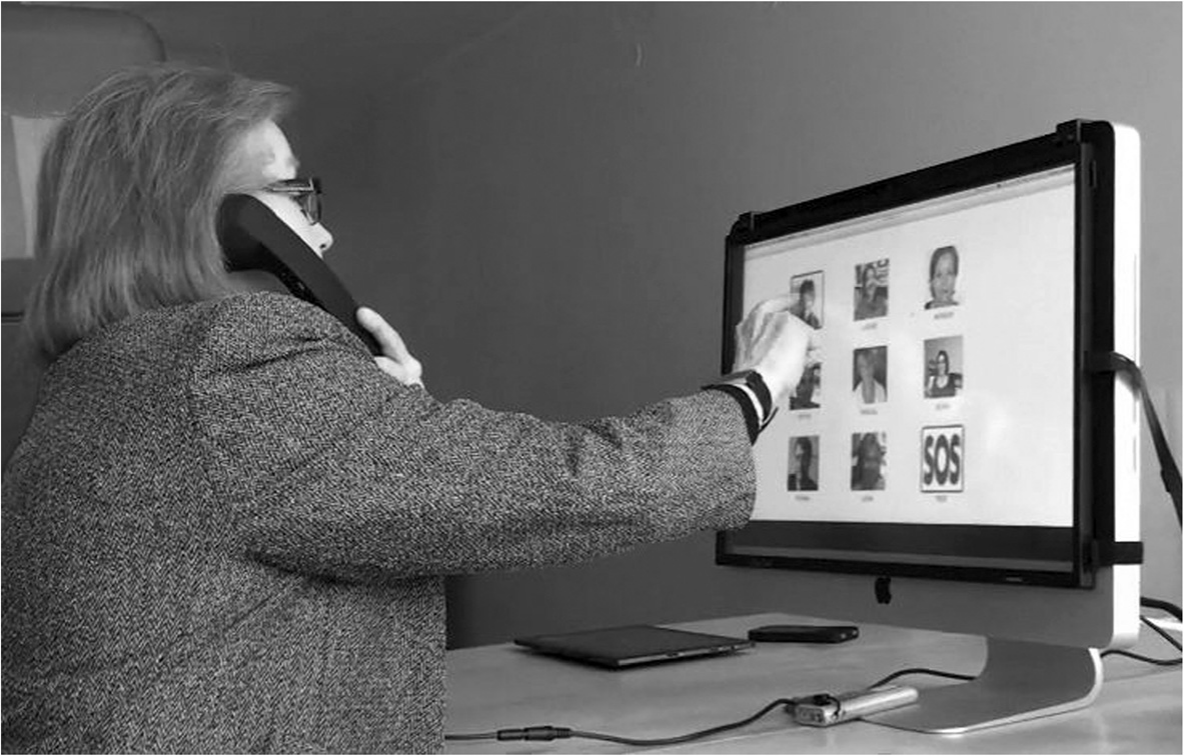
The videophone mock-up.

In order to handle video calls and conversations, an iMac computer was connected to the Internet and a video chat programme. Initially, an Android solution was used but the sound quality failed. This concept was aborted because it would be too time-consuming and expensive to make it workable. Instead the “bricks” were used as they were easy to assemble in the iMac environment by using the Apple Script facility. The “bricks” also made it possible to control many of the standard programmes on the Mac. The keyboard, the mouse, and all the cables connected to the computer were hidden, so that the participants saw a 21.5 inch screen and a handset to listen to and speak through. Since the iMac does not come with a touch screen, an add-on screen was placed on top of the original screen. Because of that, the pictures were not as bright and sharp as they would have been on a regular touch screen (see Figure 1).

### Ethics

The study was approved by one of the Regional Ethical Review Boards in Stockholm, with journal number 2010/1674-31/5. The participants received oral and written information about the study and both the persons with dementia and their significant others gave their written informed consent. Participation in the study was voluntary and all participants were assured of confidentiality. The participants’ names are fictitious.

### Procedure and data collection

This study had a qualitative case study design with interviews and observations [19].

### Preparatory, interviews and observations in the home

The data collection started with an interview with the participants with dementia and, if possible, also with a significant other in the participants’ homes after receiving their written consent. The interview with the participant with dementia was based on an interview guide [20] focusing on the participants’ experience of difficulties using their ordinary telephone, cell phone, and computer, if they had one, and how they responded to these difficulties. Furthermore, they were asked about experiences of having used Skype and a touch-screen display. To facilitate the interviews, everyday language was used, and the questions were adjusted to each individual’s situation.

Immediately after each interview, the participants’ with dementia ability to use their own telephone, cell phone or computer was assessed using the observation-based Management of Everyday Technology Assessment, META [21]. The META consists of 10 skill items assessing observable performance skills when using everyday technology. The participants’ use of their own everyday technology was observed and scored on the performance skill items using a three-category rating scale: (3 = no difficulty, 2 = minor difficulty, and 1 = major difficulty).

Each participant with dementia was observed and scored on the management of two or more telephone-related items. For example, they made a call to the researcher with their own cell phone (the researcher had written down her cell phone number on a note) or demonstrated that they were using their computer. The tape-recorded interviews and observations were conducted by two trained research assistants and the sessions varied between 1.5 and 2 hours. The interview with significant others focused on how they perceived the participants with dementia had managed to use their ordinary telephone, cell phone, and computer, and if they themselves had used Skype and a touch-screen display. Ingrid’s and Arne’s spouses were interviewed during the home visit, while Sven’s daughter and Maria’s friend were interviewed in the living laboratory.

### User testing of the videophone mock-up at the living laboratory

The next step was to test the mock-up. All test sessions were conducted at the Royal Institute of Technology’s living laboratory which is built as a self-contained apartment and gave the participants a feeling of being in a home rather than in a laboratory. The usability of the videophone mock-up was examined by using observations and interviews. An interview guide was developed for the test session [22]. Questions were asked on satisfaction with the mock-up and the design, and how easy the features were to use and understand in order to carry out the required tasks. In order to evaluate the features and the reliability of the mock-up and plan, the test procedure, and the filming during the test session, a pilot test was first conducted with two older adults without dementia. After the pilot test, some amendments in the interface were made; for example, the telephone number to SOS in the SOS-icon in the contact list was removed because the participants considered it confusing.

The test sessions lasted between two and three hours, including a coffee break. The first author (I-L.B.) was the test leader. The second author (S.L.) was responsible for the mock-up set-up; if any problems occurred during the test, he tried to solve them while underway. Two research assistants took care of practical matters: filming, audio-taping, and handling the mock-up. The participants with dementia were filmed and tape recorded during the test session. After each test session, the first two authors and the assistants reflected on the test session, embracing immediate suggestions for improvements of the mock-up. The reflections were also tape recorded.

The test procedure in each session consisted of six parts. First, the second author (S.L.) took a picture of the participant with dementia and her/his significant other and uploaded the pictures to the videophone’s contact list and their names were added. The pictures were instantly changed as new participants with dementia entered with their significant others. After that, I-L.B. presented the purpose of the study and gave a brief explanation of how the test would be conducted and assured herself of the participants with dementia and the significant other’s comprehension. In the next step I-L.B. showed how the features of the videophone worked and the participants could familiarise themselves with it for a short while. After that S.L. and the significant other went into a room nearby in order to make and answer videophone calls using a computer with a videophone programme, a camera, a speaker and a microphone. Next, the test session started. The participants with dementia were asked to carry out three different tasks: first, to make a videophone call to their significant other; second, to answer videophone calls from their significant others; and third, to make a call to the SOS (the mock-up was not connected to the SOS). The participants with dementia were encouraged to think aloud and verbalise their thoughts while performing the tasks focusing on the difficulties and problems that were encountered. In order to test if the interface of the videophone was easy to understand and use, the participants with dementia did not receive any further instructions on how to perform the tasks. I-L.B. took an observational attitude. However, if it was obvious that the participant with dementia, for example, did not know how to make a call or expressed a need for help, support was given, first as verbal guidance and second as a demonstration, if necessary. Finally, the participants with dementia were interviewed about the functions of the videophone, for example, if they preferred to use a handset, how they would prefer to turn on the videophone, and to end a call. The participants with dementia were encouraged to express their honest opinions and not hold back anything to please the researchers.

### Data analysis

The recorded interviews were transcribed and the video-taped sequences were transformed into text with detailed descriptions by the third author (S.S.). After that, the first author listened through the digitally recorded test sessions, watched the films, and read through the text several times in order to gain a good comprehension of the content. Thereafter, the text was coded by using a content analysis approach [23]. Examples of codes were: “possible to adjust individually”, “contact list”, “solution for bright light”, and “handset or not”. The codes were given names close to the participants’ own wording. As the analyses proceeded, they were compared and merged with codes from all four test sessions and participants. This meant that codes with similar content were merged into categories. To make sure that the categories were grounded in data, they were compared with data from the test sessions. The emerging findings were critically examined throughout the process by the last author (L.N.) and the third author (S.S.) in order to check the relevance and validity of the findings.

To complement the data from the test sessions, the META provided information about the participants’ with dementia performance skills using their own ordinary telephone, cell phone, and computer. This added information about their overall ability to use information and communication technology, ICT. The META items “Turn a button or knob in the correct direction” and “Follow instructions given by automatic telephone services and answering machines” were not applicable and therefore were not included in the presentation in Table 2.

**Table 2:** Results of the observational META assessment of participants with dementia*

		**Identify and separate objects**	**Identify and separate services and function**	**Perform action in logical sequence**	**Managers series of numbers/letters**	**Choose correct button or commando**	**Use appropriate force, tempo, and precision**	**Coordinate different parts of a technology**	**Identify information and respond adequately**
Ingrid	Cell phone/answer	3	2	1	na	1	3	na	2
	Cell phone/call	3	1	1	1	1	3	na	1
Sven	Cell phone/answer	3	3	3	na	3	3	3	3
	Cell phone/call	3	1	1	1	1	2	na	1
	Push-button telephone/call	3	1	3	3	3	3	3	1
Maria	Cell phone/call	3	1	3	3	3	3	na	3
	Cell phone/telephone book	3	2	2	na	2	3	na	2
	Portable telephone/call	3	3	3	3	3	3	na	2
	Computer/e-email	3	1	1	na	3	3	na	1
Arne	Cell phone/call	3	3	2	3	2	3	na	2
	Cell phone/telephone book	3	3	3	na	2	2	na	3
	Computer/find document	2	2	2	3	2	3	3	1

META = Management of Everyday Technology Assessment (Malinowsky et al. [20]).

na = not applicable. 3 = no difficulty, 2 = minor difficulty, 1 = major difficulty.

*Sequential order of the items: from easiest (Identify and separate objects) to most difficult action skills. (Identify information and respond adequately).

The results from the META provided individual maps of how they managed the action skill items when using ordinary ICT, and these were used to compare which action skills that were needed to handle the videophone mock-up.

## Results

First, the results from the interviews and observations of the participants with dementia and their significant others are presented as four cases. Thereafter, the findings from the test sessions are presented in relation to the three research questions.

### Cases

#### Case 1

Ingrid lives with her husband in a terrace house (see Table 1), and he is included as her significant other in the study. Ingrid reported that she avoids using the telephone as she has word-finding difficulties, has difficulty in expressing herself verbally, and has problems understanding what is said in a conversation. She answers the telephone only if she is alone at home. Ingrid needs help to dial telephone numbers and her husband makes all telephone calls to authorities or health care providers. She has a red cell phone that she favours because she likes its appearance, but she almost never uses it. Ingrid’s husband has written down his mobile number on the lid of that cell phone so that she can find it easily, and he helps her to ensure that it is charged. Ingrid cannot use the text and voice message functions in her cell phone. When Ingrid worked six years ago, she used a computer but she does not now. Sometimes she spends time beside her husband when he is searching the Internet for trips that they plan to do. Ingrid has never used a touch-screen technology and her husband says that she is not interested in using Skype even though she could talk to her son who lives abroad. According to the META observations, Ingrid has difficulties in knowing how to make and answer a call with her cell phone. For example, she has serious difficulties to identify and choose correct functions in the cell phone, to perform actions in a logical sequence, and to dial a telephone number (see Table 2).

#### Case 2

Sven is divorced and lives alone in his apartment (see Table 1). He makes telephone calls mostly to his daughters and sometimes to neighbours and old friends. He has close contact with his two daughters, and one of them is included as his significant other in this study (see Table 1). Sven does not remember telephone numbers and relies on a telephone book. He prefers to use his stationary telephone at home because it is much easier to find than to use the portable telephone. Sven has a cell phone that he says he benefits greatly from, although he experiences some problems; for example, he cannot use pre-programmed telephone numbers and every time he is going out he tries to remember to bring a note with the daughters’ numbers. Sometimes he is able to find a daughter’s telephone number that is stored in the cell phone function’s “latest call”. Sven also has problems to remember where he has left his cell phone. He cannot use the text and voice messaging service in the cell phone. Sven has never used a computer, Skype or touch-screen technology. According to the META observations, Sven has serious difficulties to manage his push-button telephone and cell phone. For example, he has major difficulty to identify and separate functions, perform actions in logical sequences, dial a telephone number, choose a correct command, identify information, and respond adequately when he makes a call with his cell phone (see Table 2).

#### Case 3

Maria lives alone in her apartment (see Table 1). Maria has many friends and one of her closest friends is included as her significant other in this study (see Table 1). Maria has a stationary telephone, a portable telephone, and a cell phone. She tries to keep track of the portable telephone and the cell phone. When the telephone rings she tries to find it, but very often the answering machine starts before she finds it. Maria remembers the numbers to her significant other and friends whom she talks to every day. If she is going to call someone else, she will have to look in the telephone book in her cell phone. Maria cannot use the text and voice message programme in her cell phone. She uses the computer for e-mailing, Facebook, and to check her bank account. Maria has a person that takes care of her private financial economy. She has used touch-screen displays in the hospital, but she has never used Skype. According to the META observations, Maria had some difficulties to make a call with her cell phone and to send an e-mail from her computer (see Table 2).

#### Case 4

Arne lives with his wife in an apartment (see Table 1) and she is included as his significant other in the study. They have two stationary telephones at home. Arne answers the telephone if he is alone at home. Arne’s wife makes all telephone calls to authorities and health care providers, but he participates via the other telephone so he can interfere if he wants. Arne has an old cell phone (approximately eight years) which is very important to him. If Arne wants to call someone, he uses his cell phone, i.e., not the stationary telephone. He uses pre-programmed numbers in his cell phone, but he cannot use the text and voice message programmes. He also uses the cell phone to see what time it is. Arne has never used Skype or a touch-screen display. According to the META observations, he has some difficulties to use his cell phone and computer. For example, he has difficulties to choose correct buttons and perform actions in logical sequences when making a call with his cell phone and also when trying to find a number in the telephone book in his cell phone and a document in his computer (see Table 2).

### Participants’ with dementia views on the videophone mock-up

#### *Question 1. How did the participants perceive that the features of the videophone mock-up could support them to carry out the required tasks in the test session?*

The first time the participants with dementia should try to use the mock-up, they had difficulties to understand how to start to use it and to answer and end calls. In order to make it clearer how to start to use the mock-up, the participants with dementia were informed that they could use the handset or tap the screen. Sven, Maria and Arne chose to tap the screen, and Ingrid chose to use the handset. Moreover, it was not easy to understand how to answer a call. The observations showed that most of the participants with dementia did not know how to carry out this operation when they used the mock-up in the first task, and they had to ask how it should be done. Ingrid used the handset to make a call to her husband and she got very confused as she thought that the dialling function was in the handset and she did not know how to proceed. The first author showed her how to use the contact list and the next time when she made a call she carried out the operations independently. Ingrid said that she appreciated that making a videophone call required a minimum number of steps. Arne was the only participant who spontaneously tapped the picture of his wife on the screen in order to make and answer calls. Ingrid pointed out the importance of receiving feedback when an action has been done, for example, when a call is connecting. Sven said that it could be useful to receive both visual and audio feedback.

Furthermore, it was not clear how to end a call, and most of the participants with dementia asked how they should do this. They were asked how they would like to end a call, and then all of them tapped the screen spontaneously in order to end the call. In order to make it clearer how to end a call, Ingrid’s husband suggested a red button with some pressure resistance. Ingrid liked his suggestion since a button gives clear feedback that an action has been done. However, Arne did not want to have buttons, and he said that it would be easiest to use the handset to end a call. But after some time, he changed his mind and said that it was easier to tap the picture of the person that you are talking to and draw it into the corner of the screen so that the picture disappears. Sven said that if he used the handset, it would be necessary for him to have a voice message to remind him to hang up the handset to end the call.

However, the next time, i.e., in the second task, they could carry out the tasks without difficulty.

#### Technical problems

Sometimes there were technical problems during the test session, and the participants with dementia could see a video of themselves in the left corner on the screen. We had decided not to use this function which is common in standard video communication programmes, but sometimes it still appeared; every time it happened, the participants with dementia got confused and asked why they suddenly could see themselves on the screen.

#### *Question 2. How did the participants perceive that the features of the videophone mock-up could meet their requirements?*

Overall, the participants were satisfied with the features of the videophone mock-up. One of the significant others said: “The mock-up looks like Skype but it is much easier to use”. However, they also asked for other solutions as well as the possibility to adjust the videophone individually. All participants said that they would like to have the videophone at home. However, Arne said that it is difficult to point out the gains of the videophone before you have used it in real life*.* They said that being able see each other made it easier to find topics for conversation. Sven, for example, said that it was funny and exciting that the person you talk to can see you in your own environment and perhaps that would help you to come up with something more that you would like to talk about. Moreover, Maria said that video communication provides more stimulation than a regular telephone call. She stated: “The videophone feels meaningful and fun. There are other things that are useful, but the videophone also feels good”. Moreover, Ingrid’s husband pointed out that if the person with dementia can handle a call independently without being afraid of making mistakes, he or she may feel competent and enjoy using the videophone. Moreover, he said that if Ingrid had a videophone she might regain the joy of calling her friends again.

#### Options

**Adjust individually** The participants with dementia pointed out that it should be possible to adjust the features of the videophone to each individual’s needs and wishes. For example, Sven pointed out that it is important that the user can adjust the volume and select a ring tone that is familiar. Maria expressed a need to call persons without using the video technology. She was comfortable with using the contact list in her mobile phone; therefore, she said that it could be a good idea to have a picture in the contact list containing a dialling function. Furthermore, she would like to have a picture in the contact list containing an address book. However, the other participants with dementia pointed out that it must be possible to see all contacts on one page on the screen because it is too difficult for persons with dementia to select a contact from a list.

**Auto-brightness** During the test sessions we had some problems with the light in the test room nearby, where significant others and S.L. operated the communication with the participants with dementia. It was difficult for the participants with dementia to see the video on the screen, i.e., their significant other, as he/she should be, because the test room was either too dark or too bright. When it was too sunny we had to close the curtains and set up a lamp behind the mock-up; when it was too dark the lamp was turned on.

**Handset** The participants with dementia agreed that it should be possible to have a handset if this was the best solution for the user. Sven said that he would like to have a handset because he was used to it. He said that he would probably look for a handset even if he had chosen not to have one. Ingrid thought that it was more comfortable to use a handset. Arne assumed that the handset could be useful if you want to talk in privacy or if you have hearing problems. Based on the difficulties to know how to handle the handset that we observed, it is obviously important that the hanging up function for the handset is clear and intuitive and that the videophone could give feedback if something goes wrong.

**Contact list** Ingrid said that the contact list with the picture dialling function was easy to use. However, Arne had some ideas about how the contact list could be improved to be easier to use. He said that having a red frame around both the SOS picture and the picture of a significant other could be confusing. Arne suggested that the red frame should only appear around the SOS picture. Instead, the frame around one of the significant others could be green he said. Moreover, he suggested that the SOS picture should be a little bit bigger than the other pictures in order to prevent the user calling SOS by mistake.

**Call history log** Maria wanted to have a function that she could use to check her most recent calls in order to find out if she had called someone or not. Arne assumed that a log or a voice message that could tell the user if he or she had made a call recently could be useful. Some of the significant others also expressed a need for a log, and one of them wanted the possibility to tape record the conversations so that he could check on them later.

**Camera** Ingrid pointed out that it is important that the user can choose *when* he or she would like to use the camera. But Sven said that it could be too difficult for the persons with dementia to remember how turn the camera off. He said that it would be easier to use the ordinary telephone when the user did not want to use video technology. Some of the significant others suggested that instead of turning the camera off, it should be possible to choose to have a still picture on the screen. Sven’s daughter mentioned that it could be too difficult for Sven to remember to turn the camera on and off, and she said that her father first of all needed a telephone with a picture dialling function.

**Size** The participants with dementia said that the size of the videophone mock-up was big, but only two of them said that it was too big.

**Touch screen** The participants with dementia said that it was easy to learn how to tap the touch screen. But Ingrid’s husband thought that it could be more abstract to learn to tap a screen in order to end or make a call. He said: “Even if we think it is easy, it is new learning for the persons with dementia”.

**Voice message** Ingrid and her husband as well as Sven expressed that they would like to have a voice message and a picture on the screen of the person that is calling when the videophone is ringing. Sven’s daughter said that a voice message is better than a text message since the latter could be very stressful to persons with dementia. Sven had difficulties to understand text messages which resulted in feelings of stress; every time that he was stressed he lost control and made several mistakes, she said.

### Results of the observational META assessment

The META items “Identify information and respond adequately” and “Identify and separate services and function” were the most difficult items to manage when the participants with dementia used their own cell phone, but this was not observed to be difficult when they used the videophone mock-up once they had understood how to use it. Ingrid and Sven had problems to manage phone numbers when they used their own cell phone; when they used the picture dialling function in the videophone, they did not need to manage numbers. Ingrid said: “It was very easy to just tap the picture when you would like to call someone and you do not need to remember the number”. For a description of the META results see Table 2.

## Discussion

The findings from the test sessions showed that the videophone mock-up was perceived as easy to use when making and answering phone calls, after once having understood the basics. Moreover, the participants with dementia thought that it was enjoyable to use and all participants said that they would like to have the videophone at home if it was possible.

However, the design of the videophone was not yet optimal, even if the design process had been carried out according to the inclusive design approach [17]. The needs of the target group had been well documented in the form of a requirement specification and a design concept that had been evaluated using focus groups with health care professionals, persons with dementia, and their significant others [1,12]. There were some challenges translating these requirements into technical functions in the videophone mock-up. Therefore, the design of the videophone had flaws and sometimes technical problems occurred that caused confusion for the participants with dementia. Moreover, the design did not provide information on how to make and answer a call. The participants with dementia had difficulties to know how to do this, and thereby guidance was necessary. There are researchers who argue that persons with dementia should not be involved too early in the development process of new technology, since exposure to poorly functioning technology can bring experiences of failure and disappointment [24,25]. This risk has to be taken into consideration in relation to the results that can be achieved if the target group is not involved in user tests [5]. A decision was made to do the testing in a laboratory setting in order to be very clear that the product was not yet ready for use in the home. Albeit facing some problems in the test sessions, the participants with dementia strongly expressed positive feedback and appreciation of being involved. However, it is important that new technology is reliable and works as expected when actually put to use in real life situations; if not, it could have a negative influence on the willingness to use the product even if it would be improved [25].

The participants made some suggestions for improvements on the videophone. They pointed out that the design should have a flexible design that is easy to adjust to each user’s needs, habits, interests, and abilities. If the videophone has a flexible design, it could also be useful for other groups, for example, people with brain injuries and older adults. However, if it has too many optional functions, it may be difficult for the users and their significant others as well as for therapists who prescribe assistive technology to identify and choose which functions are needed to make individual adjustments [26,27]. Therefore, it is important that the videophone is easy to customise for individuals and their changing needs in order to facilitate its use [12].

Interestingly, the participants with dementia knew intuitively how to tap the touch screen. However, it is not known if they would have remembered how to do it later on. Other studies have found that persons with dementia almost always can use touch screens [4,28,29]. Touch screens seem to provide a direct sense of feedback and affordance so that persons with dementia can use them independently or with encouragement and appropriate prompting [4], suggesting that our design concept based on a touch screen is a promising one.

The picture dialling function was perceived as very easy to use according to the participants with dementia, as they did not need to remember, retrieve or dial telephone numbers. This is in accordance with a study of the COGKNOW Day Navigator which was equipped with a picture dialling function and was perceived as helpful by the persons with dementia who participated in the study [5]. Furthermore, Perilli and colleagues [9] found that pictures could help persons with dementia recognise persons that they would like to call more rapidly and with more certainty. Moreover, our findings exemplify that the participants with dementia spontaneously could express how the design of the videophone could be improved. Interestingly, one of them tried to find a solution for how to finish a call, and he was able to verbalise his ideas. In the literature it has been pointed out that persons with dementia might need extended time to assimilate impressions, and they often have difficulties to express their needs and verbalise ideas due to working memory impairment [2,4]. Accordingly, testing must be adjusted to the participants’ with dementia prerequisites, and our set-up of test sessions seemed to provide the necessary circumstances for participants with dementia to reflect on their experiences and give creative suggestions for the tested product.

The participants with dementia underscored that they wanted to decide if the camera should be used during the video call. In an earlier study within this design project, the participants with dementia also emphasised this issue, while the significant others wanted to monitor the person with dementia in order to check that everything was well. Moreover, one of the significant others wanted to have the possibility to tape record the conversations so that he could check on them later [12].

This points out a dilemma in the situation where significant others often have worries about the person with dementia; therefore, they would like to use a home monitoring system in order to check that everything is okay. Systems for monitoring might have important benefits for emerging situations and increase safety and security to support persons with dementia to stay independently in their own homes [30]. However, when technology is brought in as support in everyday life for persons with dementia and their significant others, it is important not to misuse technology, and the ethical aspects should always be acknowledged [31,32].

There are several ethical and methodological aspects to consider when involving persons with dementia in research [33]. The participants with dementia in this study participated together with their significant others, and it was important to find appropriate ways to collect data so that the information gathered represented the view of the persons with dementia and not only the opinion of their significant others [5]. A strategy in this study was to try to establish a good relationship, create a relaxed atmosphere, and be responsive to the participants’ with dementia reactions. If the participants with dementia appeared uncertain during the test session, the researchers assisted him or her to feel more comfortable. For example, if the participants with dementia did not know how to carry out a task, the first author showed each step; after that assistance, the participants with dementia performed the operations. If the participants with dementia needed more support, the steps were shown again and guidance or prompts were given. Moreover, adequate time allowed the participants with dementia the possibility to reflect on each question at their own pace and some questions were repeated or reformulated to ensure that they were understood. However, there is a risk that the participants in this study wanted to give positive feedback on the videophone mock-up in order to please the researchers. To avoid this possibility, we underscored that the goal was to receive as many critical reflections as possible in order to develop a videophone that is easy to use by persons with dementia.

## Conclusions

In summary, the videophone mock-up was generally valued as being enjoyable to use. The META assessments showed that the participants with dementia had difficulties to use their own computers and telephones. However, these difficulties were not observed when they used the videophone mock-up. Most of the features of the mock-up were well understood and easily used during the test, partly guided by the test leader. However, it was not clear how to start to use the videophone, i.e., to make, answer, and finish a call, and the participants with dementia needed guidance. This indicates that the design needs to be further developed in order to be easy to use for persons with dementia. Nevertheless, the joy of being able to use the videophone seemed to be so strong that this balanced the difficulties. Moreover, the participants with dementia were satisfied with the features of the videophone, but they also made some suggestions for improvements.

## Competing interests

The authors declare that they have no competing interests.

## Authors’ contributions

I-LB: Led on the data collection, analysis and interpretation, contributed on design of the product, wrote the first draft of the manuscript. SL: Contributed to the design of the product, data collection and critical revision of manuscript drafts. SS: Contributed to data collection and critical revision of manuscript drafts. LN: Lead for the project, led on the conception and design, supervised the researchers, and critical revision of manuscript drafts. All authors read and approved the final manuscript.

## Pre-publication history

The pre-publication history for this paper can be accessed here:

http://www.biomedcentral.com/1471-2318/14/49/prepub
